# Formulation and nutritional evaluation of weaning food processed from cooking banana, supplemented with cowpea and peanut

**DOI:** 10.1002/fsn3.51

**Published:** 2013-07-16

**Authors:** Francisca I Bassey, Kay H Mcwatters, Christopher A Edem, Chukwujindu M A Iwegbue

**Affiliations:** 1 Department of Pure and Applied Chemistry, University of CalabarP.M.B. 3661, Calabar, Nigeria; 2 Department of Food Science and Technology, University of Georgia1109 Experiment Street, Griffin, Georgia, 30223-1797; 3 Department of Chemistry, Delta State UniversityP.M.B. 1, Abraka, Nigeria

**Keywords:** Cooking banana, legumes, weaning food

## Abstract

The possibility of processing a ready-to-eat nutrient-rich weaning food (WF) for infants within the age group of 0.5–0.9 years from cooking banana fortified with popular and affordable legumes (cowpea and peanut) was investigated with the aid of computer software and available technology in Nigeria. A composite of 47% cowpea, 40% ripe banana, and 13% peanut was processed, analyzed to compare the actual nutrient composition to that predicted by the software and that of two popular commercial WFs produced by Gerber Products Company: rice with banana (RB) and oats with banana (OB). Proximate composition was determined by Association of Official Analytical Chemists (AOAC) methods, in vitro digestibility by the pH drop method, and amino acid was determined using high performance liquid chromatography. Essential amino acid values were comparable to the predicted values. Protein and oil contents had values of 16.89% and 8.38%, 6.9% and 1.10%, and 12.03% and 3.16% for WF, RB, and OB, respectively. Octadecenoic (oleic) acid had the highest value of 3.65% followed by octadecadienoic (linoleic) acid with a value of 2.64% amounting to 76.69% of the total fatty acid. Total sugar content of WF was recorded as 15.96 g/100 g, with fructose having the highest value of 8.07 g/100 g, followed by dextrose with a value of 7.66 g/100 g. In vitro-digestibility was in the order OB>WF>RB. The results show that it is feasible to produce precooked WF which has the potential to meet the nutritional needs of an infant, from local staples using computer-assisted technique and inexpensive technology available in Nigeria.

## Introduction

The weaning period is a crucial period in an infant's life. At the age of 5–6 months, most infants begin to eat supplementary semisolid foods. At this stage homogenized infant foods play a major role in their nutrition (Martinez et al. 2004). Weaning foods (WFs) for a child in a developing country like Nigeria where WFs are relatively expensive is out of reach of a majority of the people and may result in malnutrition and pose a risk to the life of a child, particularly if the parents are low-income earners. Most WFs commonly sold in Nigeria are composed mainly of cereal grains which contribute about 42% of the total daily calories and 49% of the total daily protein (Keshinro et al. 1993). The maize (corn)-based products are usually in the form of porridges such as pap. Wet sieving and steeping have considerable effects on the protein losses in pap resulting in pap being a poor WF for infants (Banigo and Muller 1972). This loss of an appreciable proportion of original nutrient contents of pap during its production has led to the development of soy pap. Soybean is often in short supply in some regions and out of the reach of the low-income population; this has led to an increase in the cost of production of soy-based WF, therefore reducing its affordability (Agbede and Aletor 2004). Emerging evidence (Baker 1994) indicates that diseases such as hypertension, cardiovascular diseases, respiratory diseases, and diabetes are related to poor health and nutrition of the infant; thus, the need to provide a low-cost, nutritious weaning supplement for infants cannot be overemphasized. Baker (1994) argues that malnutrition during infancy permanently changes the body's structure, physiology, and metabolism, leading to coronary heart disease and stroke later in life. There is therefore the need to explore the nutritional potential of affordable, alternative carbohydrate- and protein-based food crops such as cooking banana (CB) (*Musa acuminata*) and legumes. How infants are fed appears to influence their long-term development and health (Baker 1994), thus heightening the importance of improving infant food.

This article therefore investigates the use of CB supplemented with common legumes (peanut [PN] and cowpea [CP]) in the formulation of WF. Banana is fourth on the developing world's list of food crops and a major staple food for millions of people throughout the tropics. It can be processed in many ways such as cooking, boiling, steaming, frying, roasting, or can be dried and milled into flour. In Nigeria, banana is often grated and cooked into porridge (Dosunmu and Bassey 2003).

Bananas are often the first solid food fed to infants (Dosunmu and Bassey 2003). Gowen (1995), however, noted that in preparing food where children are the primary target, it is especially important that a CB diet be complemented by other foods of greater energy and protein as the weaning period is a crucial time in an infant's life. In view of the urgent need for protein sources to combat malnutrition in the tropical countries, screening efforts for new crops have focused more on potential sources of concentrated proteins (FAO 1964). CPs and PNs are legumes of major dietary and economic importance (McWatters et al. 1995). They are favored because of their palatability and versatility in the preparation of food, contribution to nutritional status, and low cost as protein source compared to animal protein (McWatters et al. 1995). Flours from both legumes have unique physicochemical, functional and sensory properties and have potential for use as ingredients in composite flour mixtures (Muego–Gnanasekharan and Resurreccion 1992; Prinyawiwatkul et al. 1994; McWatters et al. 1995; Yeh et al. 2002). In this paper, we describe the processes involved in formulation of a novel WF from CB supplemented with CP and PN by the aid of formulation software. The nutritional characteristics are evaluated and compared with that of two popular WFs (oats with banana [OB] and rice with banana [RB]) produced by Gerber Products Co. (Fremont, MI).

## Material and Methods

Forty kilograms of ripe CB, 36 kg of black-eyed CP, and 23 kg of lightly roasted PN were purchased from International Farmer's Market in Lake City, Twelve Baskets in Mableton and Tara Foods in Albany, respectively, all located in Georgia, U.S.A. Extensive preliminary trials were carried out to determine the appropriate processing methods to be employed for each of the food components. Samples of commercial WF, OB, and RB (Gerber Products Co.) were purchased from the local Kroger grocery store at Griffin, Georgia, and used as reference samples.

### Treatment and drying of banana

**Figure 1:**
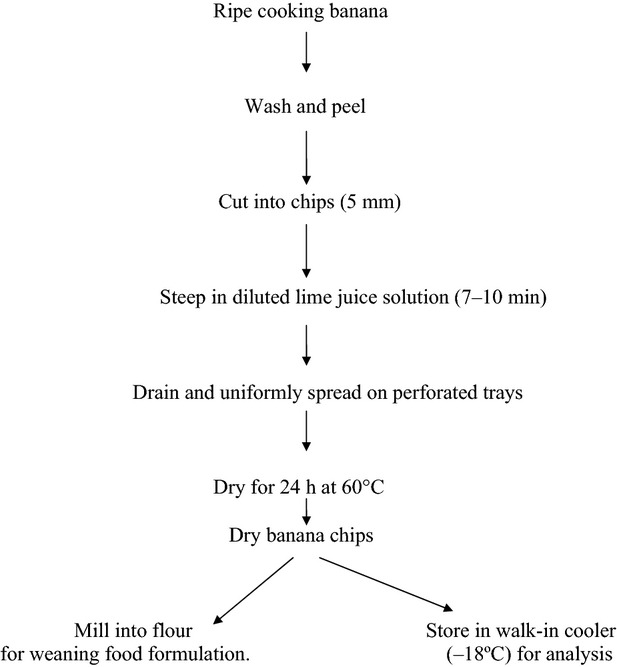
Flow chart for the processing of cooking banana.

Banana was purchased in two batches to prevent overripening and spoilage. Each batch was further divided into two lots for processing. Each lot was weighed, washed, peeled, sliced, and steeped in aqueous lime juice (100 mL lime juice to 500 mL water) for 7–10 min to prevent enzymatic browning. It was then drained, spread on perforated aluminum trays, and dried in a forced air oven for 24 h at 60°C. Some of the products were milled into flour with the use of a 4 E Grinding mill (Straub Co., Hatboro, PA) and stored in a USD-4 SN 61531-T walk-in storage chamber (N. A. Brown and Sons, Friona, TX) at −18°C until used (Fig. 1).

### Cooking, drying, and decortication of CP

**Figure 2:**
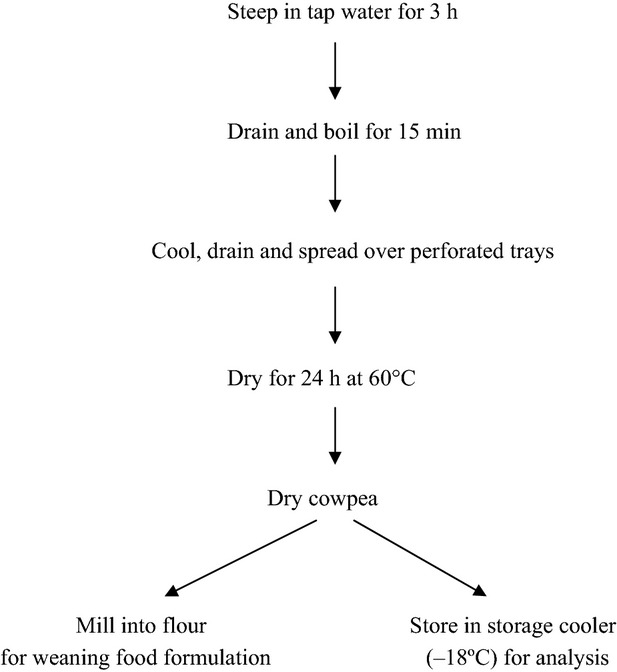
Flow chart for the processing of black-eyed cowpea.

CP (36 kg) was divided into seven portions for processing. Each batch was steeped in excess tap water at room temperature (25°C) for 3 h, drained, and then poured into boiling water in a steam-jacketed kettle. The CP was allowed to boil for 15 min. Thereafter, the steam was replaced by cold water which was passed through the jacket to stop further cooking of the CP. Cooling was continued until all the CP was removed from the kettle. The CP was dried in a forced air oven for 24 h at 60°C on perforated aluminum trays. The dry CP was then passed through a 4 E Grinding mill (Straub Co.) that had the clearance between its plates adjusted to separate the cotyledons from the seed coats with minimum grinding of the seed. Decortication of the seeds was carried out with the aid of an Alamco air blast seed cleaner (Allan Machine Co., Ames, IA). The receiving pan of the seed cleaner was positioned directly under the plates of the mill to receive the loose cotyledons while the seed coats were being winnowed away. A portion was milled into flour with the aid of the grinding mill with the clearance between the plates adjusted to enable adequate friction. The grinding mill was used in preference to other mills since this simulates available technology in Nigeria and West Africa as a whole. Both portions were kept in labeled airtight containers and stored at −18°C in a USD-4 SN 61531-T walk-in storage chamber (N. A. Brown and Sons). The remaining lots were processed and stored using the procedure described above. Two lots were processed weekly (Fig. 2).

### Peanuts

The PNs were sorted and the overroasted/defective seeds discarded. A portion of the PN was coarsely chopped into chunks with the aid of an Oster 6634 food processor (Sunbeam Products Inc., Delray Beach, FL) and stored in an air tight container at −18°C.

### Formulation of WF

A ready-to-eat WF was designed with the aid of “ESHA (Elizabeth Stuart Hands and Associates ESHA Research Inc., Salem, OR) Food Processor and Nutrient Analyser” computer software. Several trial formulations were proposed and evaluated by the ESHA software. A composite of 47% CP, 40% ripe banana, and 13% PN was found to give the best nutrient and amino acid profile to meet the dietary requirements of the 0.5–0.9 years old.

The WF was prepared in batches within a period of 6 days; this was done in order to meet the Food and Drug Administration (FDA) requirements for food analysis. The appropriate amounts of CP, ripe banana, and PN were weighed, mixed, and comilled with the aid of a 4 E Grinding mill (Straub Co.). The resultant product was fed back into the mill and the clearance between the plates further adjusted to allow more friction between the plates. The comilled product was passed through the mill four times and each time the clearance between the plates was further reduced until the desired particle size was attained. All the portions processed for the 6 days received the same treatment. The different portions were combined and mixed with the aid of a ribbon mixer (Model HD1½-3SS; Munson Machinery Co., Inc., Utica, NY) to obtain a homogeneous flour. The WF was stored in sealed containers at −18°C in the USD-4 SN 61531-T walk-in storage chamber (N. A. Brown and Sons) for analysis.

### Proximate composition

Recommended methods of the Association of Official Analytical Chemists (AOAC 1995) were employed in the analysis of moisture, fat, dietary fiber, and ash contents.

### Moisture

An Isotemp vacuum oven (model 281A; Fisher Scientific, Pittsburgh, PA) was used to determine moisture content. Three grams of sample was weighed into a predried aluminum weighing dish and covered with a lid. This was then placed in the oven for 14 h with the temperature and pressure kept at 105°C and 25 mmHg, respectively. The sample was transferred to a dessicator with the aid of a Kimwipe to cool and then weighed. The residue was recorded as total solids and stored in a desiccator for fat analysis while percentage moisture was calculated from loss in weight by the following formula:


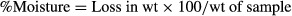


Each sample was analyzed in triplicate.

### Fat

Fat was determined by petroleum ether extraction using the Goldfisch apparatus (model 35001; Laboratory Construction Co., Kansas City, MO). Predried and labeled oil extraction beakers were weighed. Two grams of moisture-free sample was weighed into numbered, labeled oil extraction thimble and glass wool placed at the top of the thimble to keep the sample in place during the extraction process. The thimbles containing the sample were placed in glass sleeves and the sleeves placed into the prelabeled extraction unit. The oil extraction beakers were filled with 50 mL of petroleum ether and attached to the unit. The cold water was turned on followed by the power switches. The unit was allowed to reflux overnight. With the switches turned off, the glass sleeves were replaced by solvent reclaiming tubes and the system allowed to run for a few minutes until the level of liquid in the oil extraction beakers reached 10 mm. The beaker was removed and transferred to a vacuum oven for 2 h. It was then taken out and placed in a dessicator to cool and the weight of oil determined. Oil content was calculated by the following formula:


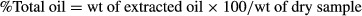


Each sample was analyzed in triplicate.

### Ash

A muffle furnace (FA 1730; Thermolyne Sybron Corporation, Dubuque, IA) was used to determine the ash content of the sample. Into preheated, cooled, and weighed crucibles, 1 g of sample was placed. The crucibles were placed in the furnace and left overnight with the temperature set at 525°C. The furnace was then turned off and the temperature allowed to drop below 250°C before the samples were transferred to a dessicator, cooled, and weighed. The percentage ash was calculated as:


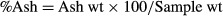


Each sample was analyzed in triplicate.

### Protein

Nitrogen content was determined using the Leco Nitrogen Analyzer (model FP 2000; St. Joseph, MI) which is a nondispersive, infra red, microcomputer-based instrument. Sample (0.2 g) was weighed into the sample boat and the weight registered on the attached computer. The key designated as “analyze” was selected and the sample pushed into the combustion chamber. The furnace and the oxygen gas caused the sample to combust releasing nitrogen gas and an oxide of nitrogen. The nitrogen content was recorded and the protein content calculated using a conversion factor of 6.25 for CP and banana while 5.46 was used for PN. Three replicates of each sample were analyzed.

### Carbohydrate

This was calculated by difference (i.e., 100 minus sum of percentages of moisture, ash, lipid, and protein).

### Calorie

One of the methods specified by FDA was employed. This uses the general factors of 4, 4, and 9 calories per gram of protein, total carbohydrate, and total fat, respectively, to calculate the calorie content of food.

### Fatty acids

Fatty acids were determined by gas-liquid chromatography with flame ionization detection (GLC-FID)/capillary column based on the method used by Oliveira et al. (2000).

### Vitamins

Vitamins A and E were analyzed using the high performance liquid chromatography (HPLC) method described by Lee et al. (2000). Ascorbic acid was extracted with metaphosphoric acid and acetic acid and quantified by fluorometric analysis according to the method of AOAC (1995). All samples were analyzed in triplicate.

### Sugars

Sugars were analyzed by HPLC as described by Linden (1995).

### Dietary fiber

Dietary fiber was determined according to procedure 985.29 of AOAC (2001).

### Statistical analysis

Data were analyzed statistically with the aid of the Statistical Analysis Software (SAS 1990).

## Results and Discussion

### Nutrient composition of CP, PN, and CB

The results from the chemical analyses of CP, PN, and CB served as a complementary guide to the ESHA software in the preparation of the composite flour for the formulation of the WF. Moisture contents of the three samples were significantly different with values of 5.44%, 1.86%, and 3.68% for CP, PN, and CB, respectively (Table 1). The high moisture content of the CP could be attributed to the high level of water absorbed by the seeds during soaking and boiling. Ripe CB is known for its high moisture content, and 24 h drying at 60°C is not sufficient to reduce the moisture content below this level. Employing higher temperatures will have an adverse effect on the sample. PNs naturally have very low moisture content coupled with the high roasting temperature.

**Table 1:** Proximate composition and calorie content of raw materials: cowpea (CP), peanut (PN), and cooking banana (CB)

Sample	Moisture (%)	Ash (%)	Oil (%)	Protein (%)	Carbohydrate (%)	Calories (kcal)
CP	5.44 ± 0.02A	2.45 ± 0.05A	1.85 ± 0.06B	25.31 ± 0.12B	64.95 ± 0.06B	377.67 ± 0.23B
PN	1.86 ± 0.03C	2.21 ± 0.04A	51.11 ± 0.53A	27.50 ± 0.02A	17.32 ± 0.13C	639.27 ± 0.77A
CB	3.68 ± 0.02B	2.08 ± 0.05A	0.50 ± 0.05C	2.78 ± 0.14C	90.96 ± 0.14A	379.45 ± 0.50B

Data represent mean ± SD, values not followed by the same letter are significantly different (*P* ≤ 0.05) as determined by LSD.

The higher ash content of CP compared with PN and CB probably indicates a higher mineral content. Oil contents of the samples were 1.85% (CP), 51.11% (PN), and 0.50% (CB). This served as an indicator for the amount of PN to be added to the product. The protein content was 25.31%, 27.50%, and 2.78% for CP, PN, and CB, respectively, showing the need for the fortification of CB with these legumes. The carbohydrate contents were 64.95% (CP), 17.32% (PN), and 90.96% (CB) while the calorie content was 377.67, 639.27, and 379.45 kcal for CP, PN, and CB, respectively. The high calories for PN despite the low carbohydrate content indicates that fat is the major contributor here.

The pH values of the legumes were significantly higher than that of CB (*P* ≤ 0.05) and in the order CP>PN>CB with values of 6.68, 6.51, and 4.58, respectively. The high acidity of the CB could be attributed to the ascorbic acid content of the ripe fruit coupled with the aqueous lime juice solution used for steeping the banana to inhibit enzymatic browning.

### Weaning food

Table 2 shows the nutrient composition obtained for the novel WF compared with that of two commercial WFs processed from RB and OB. Carbohydrate was the predominant component of all three samples followed by protein. In the case of the novel WF, fat was the next dominant component with a value of 8.38% compared with 1.10% and 3.16% for RB and OB, respectively. From Table 2 it can be seen that the novel WF has the highest amount of protein (16.89 g) in 100 g of the food with 6.9 g and 12.03 g for RB and OB, respectively. The moisture content was observed to be in the order 4.42%, 5.32%, and 5.45% for WF, RB, and OB, respectively. Dietary fiber of the formulated food was 13.05 g.

**Table 2:** Nutrient composition of weaning food (serving size 100 g)

Sample	Moisture (%)	Protein (%)	Ash (%)	Oil (%)	Carbohydrate (%)	Calories (kcal)
WF: weaning food from our study	4.42 ± 0.03B	16.89 ± 0.21A	2.16 ± 0.11C	8.38 ± 0.01A	68.16 ± 0.38C	415.59 ± 0.53A
RB: commercial weaning food (rice with banana)	5.32 ± 0.05A	6.90 ± 0.20C	3.98 ± 0.09A	1.10 ± 0.00C	82.70 ± 0.14A	368.31 ± 0.46C
OB: commercial weaning food (oats with banana)	5.45 ± 0.13A	12.03 ± 0.05B	3.02 ± 0.60AB	3.16 ± 0.03B	76.34 ± 0.14B	381.90 ± 0.50B

Data represent mean ± SD. Values not followed by the same letter are significantly different (*P* ≤ 0.05) as determined by LSD.

The moisture content of the novel WF was significantly lower than that of the commercial products, with values of 4.42, 5.32, and 5.45 for the WF, RB, and OB, respectively. The low moisture content of the WF will have a positive effect on its shelf stability as the higher the moisture content the less stable the food will be toward oxidation reactions if other environmental factors are favorable.

The ash content on the other hand was in the order RB>OB>WF with values of 3.98%, 3.02%, and 2.16%, respectively. The high ash content of OB and RB indicates a high mineral content compared with the ash content of the formulated WF which indicates the least mineral content. The RB and OB may have been fortified with iron and other minerals which may account for the high mineral/ash contents.

A low-cost infant weaning formula based on locally available indigenous foods in India (maize and green gram) provided 11.5 g protein and 305 kcal per 80 g (Devadas et al. 1974). Gahlawat and Sehgal (1993) in their work obtained protein content in the range of 13.9–14.2% and moisture, ash, fat, and calories in the range of 5.45–6.15%, 4.20–4.61 g, 1.27–1.60 g, and 348–364 kcal per 100 g, respectively. Keshinro et al. (1993) recorded nutrient composition of ogi porridge as 1.0% protein and a caloric value of 100 kcal/100 g with no appreciable oil. Kluvitse (1999) designed two weaning formulations with the aid of computer software from maize, CP, PN, and soybean oil and obtained protein and oil content in the range of 17.5–20.0 g/100 g and 7.8–9.1 g/100 g, respectively. Chandbrasekhar et al. (1988) developed mixtures from malted ragi and horse gram and roasted PNs, which contributed 412 kcal energy and 13 g of protein. Dahiya and Kapoor (1993) reported moisture in the range of 5.37–6.16%, protein 11.7–12.8%, fat 5.08–5.98%, fiber 1.26–1.61%, ash 1.91–2.20%, carbohydrate 72.5–73%, and energy 389–392 kcal for WF processed from locally available foods. Sheikh et al. (1986) also reported 6.5% moisture and 19.4% protein in the soybean weaning mixtures they formulated. The processed WF compares favorably with these foods.

Nutrient requirements for infants up to 6 months of age were established from studies involving healthy infants who were exclusively breast fed by healthy mothers (FAO 1964; WHO 1985). The calculated energy requirements for a weaning infant ideally ranged from 414 kJ/kg per day for a 4- to 5-month old to 397 kJ/kg for the 8- to 9-month old (FAO 1973; WHO 1985). The PAG (protein advisory group) of the United Nations System recommended percentage w/w protein of 15.0% minimum if the NPU (net protein utilization) is above 80, but if it is within 60–80, a minimum level of 20% is required in every weaning/infant supplementary food (Kluvitse 1999). Fat is recommended to be (as much as feasible) up to 10% as long as it does not compromise the keeping qualities of the food; linoleic acid should be at least 1%, while ash must not exceed 5 g (Kluvitse 1999). The WF appears to meet these requirements.

Total sugar was recorded as 15.96 g/100 g, with fructose having the highest value of 8.07 g/100 g, followed by dextrose with a value of 7.66 g/100 g. The high content of fructose is probably as a result of the ripe CB used in formulation (Table 3).

**Table 3:** Sugar profile of formulated weaning food

Total sugars	15.96 (g/100 g)
Dextrose	7.66 (g/100 g)
Lactose	<0.01 (g/100 g)
Sucrose	<0.01 (g/100 g)
Fructose	8.07 (g/100 g)
Maltose	0.23 (g/100 g)

Fatty acid composition of the novel WF and that predicted by ESHA is shown in Table 4. Values predicted for some of the fatty acids did not tally with obtained values. ESHA prediction for hexadecanoic (palmitic) acid was 1.95% against 1.01%, octadecanoic (stearic) acid 0.56% against 0.22%, octadecatrienoic (linolenic) acid 0.1% against 0.29%, docosanoic (behenic acid) 0.09% against 0.2, and tetracosanoic (lignoceric) acid 0.02% against 0.13%. While values obtained for octadecenoic (oleic) acid 3.65% against 3.56% and octadecadienoic (linoleic) acid 2.64% against 2.22% are comparable. The data also showed that octadecenoic (oleic) acid which is a monounsaturated fatty acid had the highest value of 3.65% followed by octadecadienoic (linoleic) acid a polyunsaturated fatty acid with a value of 2.64% amounting to 76.69% of the total fatty acid. The relatively high percentage of unsaturated fatty acids is known to be desirable in food compared with their saturated counterparts because of their health benefits (Coultate 2002).

**Table 4:** Fatty acid profile of formulated weaning food

Fatty acid	ESHA predicted value, %	Obtained value, % (wt/wt basis)
Hexadecanoic (Palmitic)-SAT	1.950	1.010
Octadecanoic (Stearic)-SAT	0.560	0.222
Octadecenoic (Oleic)-MUFA	3.650	3.564
Octadecadienoic (Linoleic)-PUFA	2.640	2.220
Octadecatrienoic (Linolenic)-PUFA	0.100	0.285
Docosanoic (Behenic)-SAT	0.090	0.214
Tetracosanoic (Lignoceric)-SAT	0.020	0.129

SAT, saturated fatty acid; MUFA, monounsaturated fatty acid; PUFA, polyunsaturated fatty acid.

Essential amino acids of the WF were recorded as histidine 32.55 mg, isoleucine 38.88 mg, leucine 73.42 mg, lysine 59.28 mg, methionine + cystine 25.06 mg, phenylalanine + tyrosine 89.06 mg, threonine 36.86 mg, tryptophan 11.70 mg, and valine 46.19 mg, while dietary fiber, vitamin A, C, and E contents of the WF (Table 5) were 13.05%, 187 IU, 1.54 mg, and 0.84 IU, respectively (Bassey et al. 2009). The WF will need to be fortified with these vitamins. It was observed that some of the essential amino acids of the novel food were within the FAO/WHO 1989 recommended levels for 0–1 year while all were within levels recommended for 2–5 year FAO/WHO (WHO 1985).

**Table 5:** Dietary fiber, vitamins A, C, E, and iron content of weaning food (WF)

	Dietary fiber (%)	Calcium (mg)	Iron (mg)	Vitamin A (IU)	Vitamin C (mg)	Vitamin E (IU)
WF	12.78	55.9	3.0	154	1.54	0.84
Standard values^1^ (0.5–0.9 years)	19	200	0.55	400	40	4

1 Institute of Medicine (2001, 2005).

## Conclusions

A WF for infants within the age group of 0.5–0.9 years has been successfully produced from an underutilized crop, CB fortified with very popular and easily affordable legumes, CP and PN. This was done with the aid of ESHA food processing and nutrient evaluation computer software. The results obtained were close to the predicted nutrient profile though higher in some cases as was observed for protein. Available technology in Nigeria (e.g., boiling, drying, and milling) was successfully employed in the production of this food. The nutrient composition of the product was compared favorably with that of two popular commercial WFs produced by Gerber Products Company from RB and OB, respectively. The nutrient composition of the WFs was significantly different (*P* ≤ 0.05). The moisture contents of RB and OB which were not significantly different from each other were about 1% higher than that of the formulated WF. The processed WF appeared to have a higher/better nutrient profile compared with the commercial products, with a protein content of 16.89% against values of 6.90% and 12.03% for RB and OB, respectively, while the oil content was in the order 8.38%, 1.10%, and 3.16% for WF, RB, and OB, respectively. The ash content of the formulated food was significantly lower (*P* ≤ 0.05) than that of RB and OB indicating lower mineral content, but this can be improved through fortification.

It is recommended that infants fed on this formulation be breastfed for at least 2 years, as the formulation is intended to act as supplement to breast milk and a transition meal from breast milk to solid family diets and not a substitute to breast milk. Fortification of the formulation with minerals is also recommended.
